# Seroreactivity and Risk Factors Associated with Human Brucellosis among Cattle Slaughterhouse Workers in South Korea

**DOI:** 10.3390/ijerph15112396

**Published:** 2018-10-29

**Authors:** Dilaram Acharya, Seon Do Hwang, Ji-Hyuk Park

**Affiliations:** 1 Department of Preventive Medicine, College of Medicine, Dongguk University, Gyeongju 38066, Korea; dilaramacharya123@gmail.com; 2 Division of Zoonoses, Center for Immunology and Pathology, Korea National Institute of Health, Korea Centers for Disease Control and Prevention, Cheongju 28159, Korea; hwangsd@korea.kr; 3 Division of Bacterial Diseases, Center for Laboratory Control of Infectious Diseases, Korea Centers for Disease Control and Prevention, Cheongju 28159, Korea

**Keywords:** brucellosis, risk factors, slaughterhouse, serologic tests, South Korea

## Abstract

The prevalence rate of human brucellosis in high-risk populations, as well as their risk factors, have not been well understood in South Korea. In this cross-sectional study, we investigated the seroreactivity and risk factors associated with human brucellosis among South Korean cattle slaughterhouse workers. We enrolled 922 subjects working in 71 slaughterhouses across the country in 2012. A structured questionnaire was used to obtain data from the subjects, following which blood samples were collected and tested using the microagglutination test; serum titers ≥ 1:20 were considered reactive. Independent risk factors were identified using multivariate logistic regression analysis with backward elimination. Overall, 62 of 922 participants (6.7%) exhibited seroreactivity for brucellosis, and 0.4% had a seroprevalence at a dilution of 1:160. Multivariate analysis revealed that the risk factors for human brucellosis seroreactivity included large-scale slaughtering (≥100 cattle per day; odds ratio (OR), 5.41; 95% confidence interval (CI), 2.95–9.91) and medium-scale slaughtering (50–99 cattle per day; OR, 2.53; 95% CI, 1.16–5.51). Moreover, the risk of brucellosis infection was significantly lower among slaughterhouse workers who always wear protective glasses (OR, 0.27; 95% CI, 0.11–0.69) than in those who sometimes or rarely wore such glasses. Regular and consistent use of personal protective equipment, especially protective glasses, should be encouraged among cattle slaughterhouse workers to reduce brucellosis infection.

## 1. Introduction

Brucellosis is an important zoonosis caused by organisms of the genus *Brucella*. The disease occurs in various domestic and wild animals as well as in humans [1,2]. Of six pathogenic *Brucella* species, humans are mainly affected by *B. abortus*, *B. melitensis*, *B. suis* and *B. canis* [1]. Human brucellosis is an enormous public health issue with more than 500,000 new infections occurring annually worldwide [3]. Human and animal health, economic development, agricultural trade, and tourism have been greatly impacted in regions in which the disease is endemic [4,5].

Major clinical features of human brucellosis include undulant fever, weight loss, back pain, fatigue, night sweats, hepatomegaly, splenomegaly, and general aches [6,7,8]. Complications such as arthritis, spondylitis, osteomyelitis, epididymitis, orchitis, and (in severe cases) neurobrucellosis, liver abscesses, and endocarditis have also been reported [6,7]. Humans are infected through contact with carrier animals and their products; consumption of meat, raw milk, and unpasteurized dairy products; and inhalation of infectious aerosols [9,10,11]. Therefore, veterinarians, slaughterhouse workers, farmers, and laboratory personnel who are frequently in contact with animals are at risk of acquiring brucellosis [12].

Based on variations existing in context and behavioral patterns, risk factors for human brucellosis vary and are of many different kinds. For instance, human brucellosis has been found to be associated with residence in rural areas, being single, and consuming locally processed milk products in Uganda [13], older age group and veterinarians in Mongolia [14], contact with livestock and consumption of fetus and placenta in Ecuador [15], veterinary pharmacists and animal handlers, and number of years spent working with animals in India [16]. Additionally, contact with livestock, especially goats in Bangladesh [17], consumption of raw milk in Pakistan [18], regular ingestion of raw milk, exposure to goats (herding, milking, and feeding), and handling of animal hides in Kenya [19], and slaughtering pigs in the USA [20] were reported to be associated with human brucellosis. Transmissions of human brucellosis can be intervened through some specific measures such as vaccination of animals against brucellosis, provision of personal safety measures, and intervention related to food safety [21,22]. However, such specific measures depend on knowledge and awareness about the specific risk factors and status of infection.

In South Korea, human brucellosis is mainly caused by *B. abortus*, which is the principal strain that infects cattle [5]. Since the designation of human brucellosis as a National Notifiable Infectious Disease in 2000, 761 patients have been diagnosed as of July 2018, with the peak occurring in 2006 (215 cases), according to the Korea Centers for Disease Control and Prevention [23]. In 2007, Yoo et al. found that the seroreactivity and seroprevalence among cattle slaughterhouse workers were 6.1% and 0.7%, respectively [24]. While they reported that cattle blood splashed around workers’ mouths as well as bodily exposure to cattle feces and urine were associated with an elevated seroprevalence of *Brucella* in a univariate analysis, they did not perform multivariate analysis in their study. Moreover, several potential influencing factors such as the types of slaughtering activities and wearing protective glasses were not investigated. Given the paucity of evidence regarding the risk factors for brucellosis infection in South Korea, we performed this study to investigate *Brucella* seroreactivity among cattle slaughterhouse workers in South Korea, as well as its associated risk factors.

## 2. Materials and Methods

### 2.1. Study Design and Subjects

This cross-sectional study targeted all cattle slaughterhouse workers across South Korea relying on available data from the Ministry for Food, Agriculture, Forestry, and Fisheries of Korea. We enrolled a total of 1017 cattle slaughterhouse workers working in 71 slaughterhouses that were operating across Korea in 2012.

### 2.2. Data Collection

Data were collected using a self-developed structured questionnaire that was adapted from a previous study for cattle slaughterhouse workers [24]. The questionnaires consisted of four parts: (i) general characteristics of the study participants; (ii) types of slaughtering activities; (iii) work hygiene-related factors (i.e., wearing personal protective equipment, contact with blood or feces, and presence of wound on skin); and (iv) other potential risk factors (cattle breeding, and consumption of raw beef, by-products and milk). Our study team visited all the cattle slaughterhouses in June 2012. Every study participant provided written informed consent after receiving an explanation of the aims, objectives, and procedures of the study in detail before conducting the interview. Researchers also checked and verified the completeness of the questionnaires in order to ensure the quality of the collected data.

### 2.3. Serologic Testing

On the same day as the interview, blood samples (10 mL) were collected from the participants. Sera were transferred to the Korea Centers for Disease Control and Prevention to test for brucellosis. A microagglutination test (MAT), which is regularly used to diagnose human brucellosis in South Korea instead of the serum agglutination test (SAT), was performed with a commercial *Brucella abortus* antigen to quantify the amount of agglutination as described by Park et al. [25]. With reference to enzyme-linked immunosorbent assay (ELISA), the sensitivities of the MAT for IgG and IgM were 93.3% and 96.7%, respectively, and the specificities of the MAT for IgG and IgM were 96.7% and 98.3%, respectively [25]. Serum titers ≥ 1:20 as determined by the MAT were considered seroreactive, considering the low incidence rate of human brucellosis in 2011 in South Korea (0.04 cases per 100,000 persons) [23].

### 2.4. Statistical Analysis

The data were analyzed using SPSS Version 17.0 (SPSS Inc., Chicago, IL, USA). We conducted univariate logistic regression analysis to determine the significant factors associated with *Brucella* seroreactivity among slaughterhouse workers. Subsequently, variables determined to be significant (*p* < 0.05) were incorporated into a multivariate logistic regression with backward elimination to calculate odds ratios (ORs) and 95% confidence intervals (CIs). A *p*-value < 0.05 was considered significant.

### 2.5. Ethical Considerations

The Institutional Review Board of Dongguk University Gyeongju Hospital reviewed and approved this study (approval number-12-033). Informed consent was obtained from all participants prior to enrollment in this study.

## 3. Results

A total of 922 of 1017 cattle slaughterhouse workers participated in this study, resulting in a response rate of 90.7%. The participants comprised 864 men (93.7%) and 58 women (6.3%). The median participant age was 51 (range, 21–74) years, while the median length of time on the job was 11 (range, 1–51) years. Overall, 62 of the 922 participants (6.7%) exhibited *Brucella* seroreactivity. The titer cut-offs of *Brucella abortus* ranged from <1:20 to 1:640; the distribution of the 62 seroreactive participants was 36 (3.9%) at 1:20, 16 (1.7%) at 1:40, 6 (0.7%) at 1:80, and 4 (0.4%) at 1:160 and over.

### 3.1. Univariate Analysis of Brucella Seroreactivity and Potential Risk Factors

Thirty-one factors were assessed in the univariate analysis. Among them, young individuals (<45 years) had a significantly higher risk of *Brucella* seroreactivity than older individuals (≥60 years) (*p* = 0.043). Cattle slaughterhouse workers who were working in central regions had a significantly lower risk of *Brucella* seroreactivity than those who were working in southern regions (*p* = 0.034). Additionally, individuals working in large- and medium-scale slaughterhouses (≥100 and 50–99 cattle per day, respectively) were more likely to exhibit *Brucella* seroreactivity than those working in small-scale slaughterhouses (*p* = 0.020 and *p* < 0.001, respectively) (Table 1). However, the nature of slaughter activity was not significantly associated with *Brucella* seroreactivity (Table 2). Among work hygiene-related factors, cattle slaughterhouse workers who always wore protective glasses had a significantly lower risk of brucellosis infection than those who wore protective glasses sometimes or rarely (*p* = 0.004) (Table 3). Potential risk factors such as consumption of raw beef, raw by-products, and raw milk, as well as cattle-breeding, were not significantly associated with *Brucella* seroreactivity (Table 4).

### 3.2. Multivariate Analysis of Brucella Seroreactivity and Potential Risk Factors

Multivariable logistic regression data demonstrating potential risk factors associated with *Brucella* seroreactivity are shown in Table 5. All significant risk factors in the univariate analysis such as age, region, slaughterhouse scale, and wearing protective glasses were incorporated into a multivariate logistic regression with backward elimination. Slaughterhouse scale and wearing protective glasses were maintained in the final model. Of these variables, a large slaughtering scale (≥100 cattle per day; OR, 5.41; 95% CI, 2.95–9.91) and medium slaughtering scale (50–99 cattle per day; OR, 2.53; 95% CI, 1.16–5.51) were significantly associated with *Brucella* seroreactivity. Furthermore, the risk of brucellosis infection was significantly lower among slaughterhouse workers who always wear protective glasses (OR, 0.27; 95% CI, 0.11–0.69) than those who sometimes or rarely wore protective glasses.

## 4. Discussion

Our study documented both the seroreactivity of human brucellosis and its associated risk factors using nationwide data from cattle slaughterhouse workers in South Korea. We found that, overall, 6.7% of the workers exhibited seroreactivity and 0.4% had seroprevalence at a 1:160 dilution using the MAT. Our findings are consistent with those of Yoo et al., who reported a seroreactivity of 6.1% and seroprevalence of 0.7% using a 1:160 dilution via SAT among cattle slaughterhouse workers in South Korea [24]. However, the number of bovine brucellosis afflictions in South Korea has decreased from 11,547 in 2007 to 2287 in 2012 [26], and furthermore, the number of humans with brucellosis in South Korea has decreased from 101 in 2007 to 17 in 2012 [23]. Nonetheless, individuals who are in frequent contact with animals remain at constant risk of brucellosis, and many are underdiagnosed and/or underreported [7,12].

Serologic studies performed in other countries presented differing proportions of human brucellosis seroprevalences among slaughterhouse workers. For instance, a Nigerian study found that the brucellosis seroprevalence was 59.3% among butchers [27], while an Ethiopian study found that the seroprevalence in abattoir workers, as determined by the Rose Bengal plate test, was 4.7% [28]. Additionally, the brucellosis seroprevalence was reported to be 10.3% (using SAT) in Uganda and 7.9% (using ELISA) in Iran [11,29]. While the seroprevalence of brucellosis can be influenced by several factors such as the status of infection in slaughtered animals and laboratory methods employed to analyze the samples, the brucellosis seroprevalence among cattle slaughterhouse workers in South Korea may in fact be lower than those in other countries.

We found that cattle slaughterhouse workers who were working in large- and medium-scale slaughterhouses had a significantly higher risk of brucellosis seroreactivity, which could be explained by the increased possibility of exposure to *Brucella abortus* that resides in infected cattle [5]. A Tanzanian study [30] discovered that the seroprevalence of brucellosis was associated with goat, sheep, and cattle contact, which was not unexpected and is consistent with our own findings. However, we found no publications describing the association between slaughtering scale and *Brucella* infection. Among slaughtering activities, bleeding, cutting of heads, cutting of hind legs, and manual skinning were associated with higher rates of *Brucella* infection but without statistical significance. A serologic study in Iran reported that slaughtering and transportation jobs among slaughterhouse workers were not significantly associated with *Brucella* infection [29].

In our present study, 24.5% of participants always used protective glasses; the risk of brucellosis was significantly lower among this population. Previous studies among slaughterhouse workers found that the use of personal protective equipment was associated with a lower *Brucella* seroprevalence rate [11,29]. However, these studies did not investigate the role of wearing protective glasses in brucellosis prevention. Contact with the conjunctiva of the eyes is a known route of brucellosis infection [7,21]; thus, using protective eyewear ought to prevent this disease’s transmission. Workers’ oral exposure to splashed cattle blood, or bodily contamination with splashed cattle feces/urine were associated with a higher risk of brucellosis in the univariate analysis albeit without statistical significance, which is contrary to the previous study performed among South Korean cattle slaughterhouse workers in 2007 [24]. Another study in Iran found that splashing of animal secretions was not significantly associated with brucellosis seroprevalence [29]. Differences in serologic evaluation criteria and/or in working environments may influence these results. Additionally, we found that wearing a mask was not significantly associated with brucellosis seroreactivity. This result might be influenced by using cloth masks or face masks in South Korean slaughterhouse workers, which are not waterproof. Previous studies discovered that the older age group was a risk factor for human brucellosis [19,31,32,33]. Contrary to these studies, our study showed that the lower-age group of cattle slaughterhouse workers were more likely to have seroreactivity for brucellosis in the univariate analysis. However, after a multivariate logistic regression with backward elimination in the final model, the age variable was not found to be statistically significant.

We conducted and demonstrated the evidence of human brucellosis in nationwide data from cattle slaughterhouses and associated risk factors. However, there were several limitations in this study. Firstly, we used only one test MAT to investigate brucellosis which could have low specificity, and which might have caused the selection of false positive cases. Secondly, since our study did not present the bacteriological evidence or molecular-based tests, seroreactivity results might be caused by previous exposure to infection or cross-reactivity. Thirdly, we could not examine the *Brucella* infection status of slaughtered cattle that might impact the seroreactivity status among cattle slaughterhouse workers.

## 5. Conclusions

Our study revealed *Brucella* seroreactivity of 6.7% and a seroprevalence of 0.4% using the MAT among cattle slaughterhouse workers in South Korea. Additionally, a high number of cattle slaughtered per day was a risk factor for the disease, while wearing protective glasses reduced *Brucella* seroreactivity rates among cattle slaughterhouse workers. Regular and consistent use of personal protective equipment, especially protective glasses, should be encouraged among cattle slaughterhouse workers to reduce the risk of brucellosis.

## Figures and Tables

**Table 1:** Univariate logistic regression analysis of the demographic characteristics associated with brucellosis seroreactivity among cattle slaughterhouse workers in South Korea.

	Total	Seroreactivity No. (%)	OR (95% CI)	*p*-Value
Sex				
Men	864	61 (7.1)	4.33 (0.59–31.81)	0.150
Women	58	1 (1.7)	Reference	
Age (years)				
<45	272	27 (9.9)	2.55 (1.03–6.33)	0.043
45–59	505	29 (5.7)	1.41 (0.57–3.47)	0.453
≥60	145	6 (4.1)	Reference	
Education				
Middle school or less	439	28 (6.4)	0.90 (0.53–1.51)	0.683
High school or more	482	34 (7.1)	Reference	
Duration of work (years)				
<10	383	19 (5.0)	Reference	
10–19	278	23 (8.3)	1.73 (0.92–3.24)	0.088
≥20	251	20 (8.0)	1.66 (0.87–3.17)	0.127
Region				
Northern	199	12 (6.0)	0.60 (0.30–1.23)	0.166
Central	463	25 (5.4)	0.54 (0.30–0.95)	0.034
Southern	260	25 (9.6)	Reference	
Slaughterhouse scale (cattle per day)				
<50	557	18 (3.2)	Reference	
50–99	159	12 (7.5)	2.44 (1.15–5.19)	
≥100	206	32 (15.5)	5.51 (3.02–10.06)	<0.001

OR, odds ratio; CI, confidence interval.

**Table 2:** Univariate logistic regression analysis of the association between specific work activities and brucellosis seroreactivity among cattle slaughterhouse workers in South Korea.

	Total	Seroreactivity No. (%)	OR (95% CI)	*p*-Value
Stunning				
Yes	71	3 (4.2)	0.59 (0.18–1.94)	0.387
No	851	59 (6.9)	Reference	
Bleeding				
Yes	89	8 (9.0)	1.42 (0.66–3.10)	0.372
No	833	54 (6.5)	Reference	
Cutting of heads				
Yes	122	9 (7.4)	1.12 (0.54–2.34)	0.757
No	800	53 (6.6)	Reference	
Cutting of front legs				
Yes	82	3 (3.7)	0.50 (0.15–1.64)	0.254
No	840	59 (7.0)	Reference	
Cutting of hind legs				
Yes	114	10 (8.8)	1.40 (0.69–2.84)	0.353
No	808	52 (6.4)	Reference	
Manual skinning				
Yes	132	10 (7.6)	1.16 (0.58–2.35)	0.673
No	790	52 (6.6)	Reference	
Mechanical skinning				
Yes	137	7 (5.1)	0.71 (0.32–1.60)	0.415
No	785	55 (7.0)	Reference	
Chest opening				
Yes	84	5 (6.0)	0.87 (0.34–2.23)	0.767
No	838	57 (6.8)	Reference	
Evisceration				
Yes	129	4 (3.1)	0.41 (0.14–1.14)	0.086
No	793	58 (7.3)	Reference	
Body splitting				
Yes	82	3 (3.7)	0.50 (0.15–1.64)	0.254
No	840	59 (7.0)	Reference	
Carcass washing				
Yes	85	4 (4.7)	0.66 (0.23–1.87)	0.438
No	837	58 (6.9)	Reference	

OR, odds ratio; CI, confidence interval.

**Table 3:** Univariate logistic regression analysis of the association between work hygiene-related factors and brucellosis seroreactivity among cattle slaughterhouse workers in South Korea.

	Total	Seroreactivity No. (%)	OR (95% CI)	*p*-Value
Wearing protective glasses				
Always	223	5 (2.2)	0.26 (0.10–0.65)	0.004
Sometimes/rarely	686	56 (8.2)	Reference	
Wearing a protective mask				
Always	522	42 (8.0)	1.62 (0.94–2.81)	0.084
Sometimes/rarely	391	20 (5.1)	Reference	
Wearing protective gloves				
Always	730	44 (6.0)	0.65 (0.36–1.19)	0.163
Sometimes/rarely	179	16 (8.9)	Reference	
Wearing a protective apron				
Always	823	54 (6.6)	0.75 (0.35–1.64)	0.477
Sometimes/rarely	94	8 (8.5)	Reference	
Wearing protective boots				
Always	877	58 (6.6)	0.66 (0.23–1.90)	0.436
Sometimes/rarely	41	4 (9.8)	Reference	
Taking a shower after work				
Always	893	61 (6.8)	1.98 (0.26–14.82)	0.506
Sometimes/rarely	28	1 (3.6)	Reference	
Contact with blood around the mouth				
Yes (≥once a week)	472	36 (7.6)	1.34 (0.80–2.26)	0.268
No	449	26 (5.8)	Reference	
Contact with blood around the body				
Yes (≥once a week)	641	45 (7.0)	1.16 (0.65–2.07)	0.606
No	279	17 (6.1)	Reference	
Contact with feces/urine around the mouth or body				
Yes (≥once a week)	525	41 (7.8)	1.50 (0.87–2.59)	0.140
No	394	21 (5.3)	Reference	
Presence of wound on skin				
Yes (within a year)	133	10 (7.5)	1.14 (0.57–2.31)	0.710
No	783	52 (6.6)	Reference	

OR, odds ratio; CI, confidence interval.

**Table 4:** Univariate logistic regression analysis of the association between other potential risk factors and brucellosis seroreactivity among cattle slaughterhouse workers in South Korea.

	Total	Seroreactivity No. (%)	OR (95% CI)	*p*-Value
Consumption of raw beef				
Yes (≥once a week)	452	34 (7.5)	1.28 (0.76–2.15)	0.348
No	469	28 (6.0)	Reference	
Consumption of raw by-products ^a^				
Yes (≥once a week)	318	26 (8.2)	1.40 (0.83–2.37)	0.206
No	603	36 (6.0)	Reference	
Consumption of raw milk				
Yes (within a year)	16	2 (12.5)	2.00 (0.44–8.99)	0.367
No	899	60 (6.7)	Reference	
Breeding cattle				
Yes	42	3 (7.1)	1.10 (0.33–3.67)	0.878
No	856	56 (6.5)	Reference	

OR, odds ratio; CI, confidence interval. ^a^ Raw by-products mean raw liver or stomach.

**Table 5:** Multivariate logistic regression analysis with backward elimination to identify risk factors for brucellosis seroreactivity among cattle slaughterhouse workers in South Korea ^a^.

	OR (95% CI)	*p*-Value
Slaughterhouse scale (cattle per day)		
<50	Reference	
50–99	2.53 (1.16–5.51)	0.020
≥100	5.41 (2.95–9.91)	<0.001
Wearing protective glasses		
Always	0.27 (0.11–0.69)	0.006
Sometimes/rarely	Reference	

OR, odds ratio; CI, confidence interval; ^a^ All variables having *p*-value < 0.05 in the univariate analysis were incorporated into multivariate logistic regression analysis (slaughterhouse scale, and wearing protective glasses).
